# Therapeutic targeting of mitophagy in Parkinson's disease

**DOI:** 10.1042/BST20211107

**Published:** 2022-03-21

**Authors:** Shashank Masaldan, Sylvie Callegari, Grant Dewson

**Affiliations:** 1 The Walter and Eliza Hall Institute of Medical Research, Melbourne, Victoria 3052, Australia; 2 Department of Medical Biology, The University of Melbourne, Melbourne, Victoria 3010, Australia

**Keywords:** mitochondria, mitophagy, Parkin, Parkinsons disease, PTEN induced putative kinase 1, ubiquitin

## Abstract

Parkinson's disease is a neurodegenerative disorder characterised by cardinal motor symptoms and a diverse range of non-motor disorders in patients. Parkinson's disease is the fastest growing neurodegenerative condition and was described for the first time over 200 years ago, yet there are still no reliable diagnostic markers and there are only treatments that temporarily alleviate symptoms in patients. Early-onset Parkinson's disease is often linked to defects in specific genes, including PINK1 and Parkin, that encode proteins involved in mitophagy, the process of selective autophagic elimination of damaged mitochondria. Impaired mitophagy has been associated with sporadic Parkinson's and agents that damage mitochondria are known to induce Parkinson's-like motor symptoms in humans and animal models. Thus, modulating mitophagy pathways may be an avenue to treat a subset of early-onset Parkinson's disease that may additionally provide therapeutic opportunities in sporadic disease. The PINK1/Parkin mitophagy pathway, as well as alternative mitophagy pathways controlled by BNIP3L/Nix and FUNDC1, are emerging targets to enhance mitophagy to treat Parkinson's disease. In this review, we report the current state of the art of mitophagy-targeted therapeutics and discuss the approaches being used to overcome existing limitations to develop innovative new therapies for Parkinson's disease. Key approaches include the use of engineered mouse models that harbour pathogenic mutations, which will aid in the preclinical development of agents that can modulate mitophagy. Furthermore, the recent development of chimeric molecules (AUTACs) that can bypass mitophagy pathways to eliminate damaged mitochondria thorough selective autophagy offer new opportunities.

## Introduction

Parkinson's disease (PD) is a neurodegenerative disorder that leads to chronic and progressive deficits in body movement that impact the life and lifestyle of affected individuals. Clinical features of PD include tremors, slowness of movement (bradykinesia), involuntary movement (dystonia), stiffness and rigidity, leading to abnormal gait [1,2], and in advanced stages a proportion of patients develop dementia [1,3,4]. PD is considered a disease of ageing, with more than 1% of the population over 60 affected [5,6]. However, it is now clear that the underlying disease processes initiate decades prior to clinical manifestation of motor symptoms. This is exemplified by prodromal symptoms including hyposmia, constipation, mood disorders (e.g. anxiety, apathy), visual impairment (e.g. colour perception) and rapid eye movement sleep disorder that often emerge during the early pre-motor phase of the disease and can predate motor symptoms by years.

A primary pathological feature of PD is the death of dopaminergic (DA) neurons of the *substantia nigra pars compacta* (SNpc) which is reported to occur prior to the onset of motor symptoms [7,8]. At the onset of the first motor symptoms of PD, almost 30% of neurons in the SN are reported to be lost, increasing to 50% after 5 years of symptomatic disease [7]. The resultant progressive loss of dopamine in the brain can be managed by administration of levodopa, an amino acid precursor of dopamine, to control the onset of motor symptoms. Levodopa is frequently administered together with the decarboxylase inhibitor carbidopa, which acts to prevent levodopa from being metabolised outside of the brain. Whilst still the most effective available treatment for PD, levodopa/carbidopa alleviates symptoms, but do not alter disease progression. Furthermore, patient response varies and, over time, diminishes. The most hopeful prospect of developing much-needed disease-modifying drugs that slow or even stop disease progression is to identify the triggers that initiate neurodegeneration in individuals with PD. Unfortunately, no reliable biomarker(s) currently exist to diagnose pre-symptomatic PD [8], and while two centuries have passed since the ‘shaking palsy’ was first described by Dr. James Parkinson, there is as yet no cure or disease-modifying drugs for PD [2].

Mitochondria, especially in high energy demanding cells such as neurons, play a vital role in ensuring a sufficient supply of ATP. Early studies documented the chance discoveries that agents that perturb mitochondrial function; 1-methyl-4-phenyl-1, 2, 3, 6-tetrahydrodropyridine (MPTP), rotenone and paraquat, can cause parkinsonism in humans or animals [9–11]. Furthermore, mitochondrial dysfunction including a reduction in mitochondrial complex I activity is observed in brain tissue from PD patients [12,13]. Since these early studies, mitochondrial dysfunction has emerged as an underlying cause of PD. Although PD is largely (>90%) sporadic in incidence, mutations in certain genes lead to parkinsonism commonly early in life before 50 years of age [early-onset PD (EOPD)] [14–16]. These genes include *PRKN*, *PINK1*, *DJ-1* and *ATP13A2*, all of which encode for proteins that affect mitochondrial function [14–16]. Furthermore, autosomal dominant PD can result from variably penetrant mutations in genes linked to mitochondrial form and function, such as *LRRK2* (involved in basal and PINK1/Parkin mitophagy, mitochondrial trafficking and electron transport chain) [17,18], *SNCA* (mitochondrial morphology and biogenesis; encodes for α-synuclein, the major component of Lewy bodies- a primary pathology of PD), *VPS35* (mitochondrial fission and fusion) and *CHCHD2* (mitochondrial function and biogenesis) [16]. These PD genes are increasingly recognised in the complex pathogenesis of sporadic PD with genome-wide association studies revealing genetic polymorphisms in these genes underscore a risk of developing sporadic PD [16,19,20]. Thus, failure in mitochondrial function and maintaining a healthy mitochondrial network form a common theme in the pathogenesis of diverse forms of PD.

In this review, we describe how a failure of mitochondrial quality control through autophagy of damaged mitochondria (mitophagy) underlies both monogenic and sporadic forms of PD. Furthermore, we hypothesise that enhancing mitophagy may provide clinical benefit in PD and we discuss strategies for promoting mitophagy to eliminate damaged mitochondria, including with new autophagy-targeting chimeras (AUTACs), as therapeutic approaches to limit neuronal cell death and neurodegeneration.

## The role of mitochondria in the selective vulnerability of dopaminergic neurons in PD

While different neuronal subtypes may be vulnerable in the PD brain (e.g. noradrenergic locus coeruleus neurons) [21], loss of DA neurons of the SNpc ultimately leads to the motor symptoms of PD. The form and function of DA neurons enhances their susceptibility to mitochondrial dysfunction [22]. Like most neurons, these cells last a lifetime and require tremendous energy to carry out their function. Furthermore, they exhibit unique morphologies, such as extensive axonal arborisation that increases susceptibility to mitochondrial dysfunction when it occurs, leading to neuronal impairment and degeneration [22]. This is evident through the loss of DA neurons in mice administered with compounds that inhibit mitochondrial ATP synthesis, such as rotenone and MPTP (both mitochondrial complex I inhibitors), as well as 6-hydroxy dopamine [23]. Somatic mutations in mitochondrial DNA that may perturb mitochondrial function and disrupt ATP generation are also observed in PD patients with DA neurons of the SNpc exhibiting higher accumulation of these mutations compared with other neurons [24,25]. Similarly, tissue-specific knockout of *Tfam*, a mitochondrial DNA transcription factor, leads to impaired respiratory electron transport in DA neurons and PD-like phenotypes in mice [25]. Mitochondrial complex I deficiency appears to have a key role in the pathogenesis of PD, with a recent study demonstrating that genetic ablation of *Ndufs2*, the catalytic core subunit of mitochondrial complex I, in DA neurons is sufficient to induce levodopa-responsive PD-like symptoms in mice [15]. Thus, disruption of the respiratory electron transport chain and mitochondrial ATP production may be a key driver of DA neuron loss.

Mitochondria, while being primary sources of cellular ATP, generate potentially damaging reactive oxygen species (ROS) that are tightly controlled in physiological settings through elaborate cellular antioxidant *defences*. The herbicide paraquat, which is also a mitochondrial superoxide generator, has been implicated in PD, indicating that imbalances in redox homeostasis could play a role in disease pathogenesis [11]. Dopamine itself is oxidised by neuronal monoamine oxidase, leading to the generation of hydrogen peroxide. Oxidation of dopamine is slow under physiological conditions, but its rate is sensitive to the concentration of metal ions (Fe^2+^, Cu^+^), pH and oxidative stress [26]. The damaging ROS from dopamine oxidation are mitigated by cellular antioxidants including neuromelanin, which is abundant in the SN [27]. Dopamine can also be taken up by mitochondria leading to impaired function of complex I [15,28]. Thus, an intricate balance between cellular redox buffering and mitochondrial homeostasis must be maintained in DA neurons for their function and survival [22,26]. Together, the failure in these homeostatic mechanisms, coupled with impaired cellular energetics may be sufficient to initiate neuronal loss through a single or combination of cell death pathways [26,29–32]. Another emerging source of neuronal vulnerability is inflammation initiated in response to mitochondrial DNA or ROS released from damaged mitochondria [33,34]. Taken together, mitochondrial health of DA neurons, and potentially also the supporting glial cells, appears central to mitigating PD. Consequently, the selective autophagic turnover of mitochondria as a mitochondrial quality control mechanism, i.e. mitophagy, has emerged as a molecular process of key interest in the race to develop disease-modifying drugs for PD.

## PINK1/Parkin dependent mitophagy

Mitochondria are a highly dynamic organelle, which exist as a reticulum that is constantly modified through fission and fusion [35–39]. Mitochondrial damage is sensed leading to targeted fission followed by mitophagy and recycling. Cells have evolved multiple mitophagy pathways to ensure timely and efficient quality control of mitochondria under different conditions. A subset of these pathways is driven by the ubiquitin system, whereby ubiquitin moieties, arranged in specific conformations, signal for the recruitment of the autophagy machinery [40]. However, another subset of mitophagy pathways are independent of ubiquitin and rely instead on specialised mitophagy receptors that interact directly with the autophagy machinery [40].

**Figure 1.**
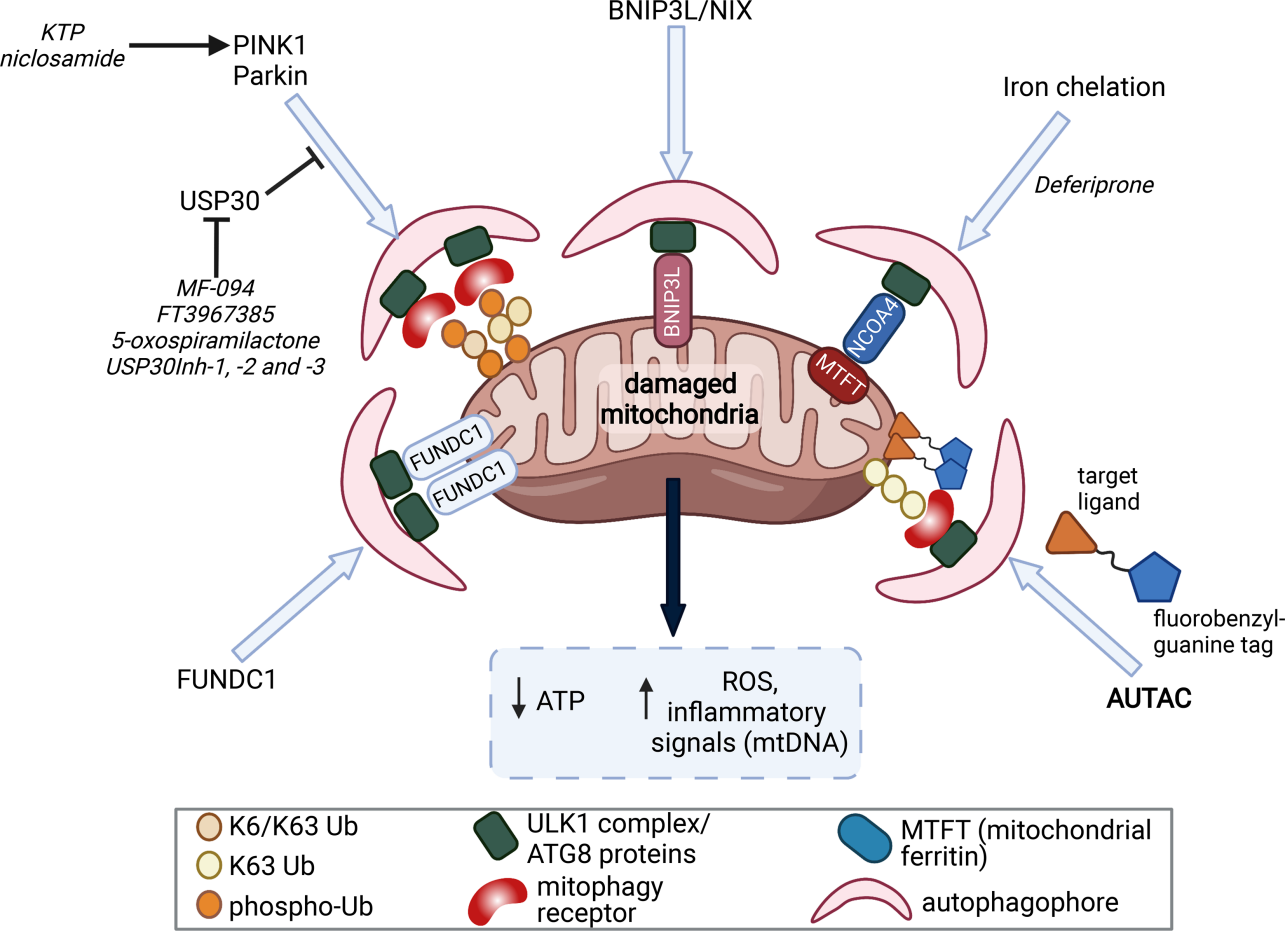
PINK1/Parkin-dependent mitophagy. Following mitochondrial damage due to protein misfolding or electron transport chain decouplers, PINK1 is stabilised on the outer mitochondrial membrane (OMM) and activates itself through trans-autophosphorylation. PINK1 then phosphorylates ubiquitin conjugated to mitochondrial proteins such as VDAC1 and mitofusin 2. Parkin translocates to sites of phospho-ubiquitin accumulation and is completely activated following its binding to phospho-ubiquitin and phosphorylation by PINK1. Fully active Parkin then catalyses the addition of ubiquitin via its catalytic cysteine (Cys431; indicated by yellow star) onto OMM proteins predominantly through K63 and K6 linkages. Ubiquitin can then be further phosphorylated via PINK1 to recruit more Parkin thus leading to a feed-forward amplification loop to mark the OMM quickly and efficiently with ubiquitin chains. This ubiquitination is opposed by the mitochondrial deubiquitinating enzyme USP30. The nascent ubiquitin chains are recognised by mitophagy receptors such as optineurin and NDP52. These mitophagy receptors recruit the ULK1 complex at focal points on mitochondria to initiate autophagophore formation with the subsequent fusion with lysosomes ultimately leading to autophagic degradation of damaged mitochondria via lysosomal hydrolases.

Mitophagy initiated by the serine/threonine kinase, PINK1 (PTEN induced putative kinase 1) and the E3 ubiquitin ligase Parkin is the most studied mitophagic process and hypomorphic mutations in Parkin and PINK1 are a common cause of autosomal recessive juvenile onset PD [32,41–45]. Following dissipation of mitochondrial membrane potential, cytosolic Parkin is rapidly phosphorylated at Ser108 in its activating element (ACT) by the serine/threonine kinase ULK1 facilitating its mitochondrial translocation and activation [46]. PINK1 is stabilised at the Translocase of the Outer mitochondrial Membrane (TOM) complex on the mitochondrial surface where it dimerises and is activated by a process of trans-autophosphorylation [47,48]. Activated PINK1 then phosphorylates ubiquitin (at serine 65) resident on outer mitochondrial membrane proteins (OMM), which serves to recruit and partially activate Parkin [41,49]. Following binding to phospho-ubiquitin, Parkin becomes fully activated via PINK1-mediated phosphorylation, at serine 65, in its ubiquitin-like (Ubl) domain [41,49,50]. Activated Parkin then decorates a variety of OMM substrates with ubiquitin. This nascent ubiquitination is a platform for further phosphorylation by PINK1 [47,51–53] to recruit more Parkin and leading to an amplification of ubiquitination through a feed-forward loop (Figure 1) [41–43,54]. An opposing deubiquitinase, USP30, anchored in the OMM, limits this process by ‘pruning’ predominantly Lys6 and Lys11-linked ubiquitin chains, thus the relative activities of Parkin and USP30 define the region of the mitochondrial network for mitophagy [55,56]. The generation of a phospho-ubiquitin ‘flag’ on the OMM recruits the autophagy receptors optineurin and NDP52, which in turn recruit the ULK1 complex at focal points on mitochondria to initiate autophagophore biogenesis with subsequent engulfment of the damaged mitochondria [44,57]. Engulfed mitochondria are ultimately degraded by lysosomal enzymes following autophagosome-lysosomal fusion [32,42], so that their components can be recycled. Hence, PINK1/Parkin replenish the mitochondrial pool to promote neuronal survival, and critically also limit the release of pro-inflammatory factors from damaged mitochondria [33]. Interestingly, while PINK1 and Parkin facilitate the clearance of depolarised mitochondria they are seemingly dispensable for basal mitophagy [58]. Furthermore, studies in Drosophila suggest that Parkin mediates the selective turnover of mitochondrial respiratory complex proteins in addition to its role in mitophagy [59]. However, the studies of Ordureau et al. [60] in human induced neurons and mouse primary neurons questioned the selective turnover of proteins by Parkin.

Independent of their role in mitophagy, PINK1 and Parkin have been recently shown to suppress adaptive immunity by regulating the production of Rab9-dependent mitochondria-derived vesicles (MDVs) [61]. In the absence of PINK1 or Parkin, MDVs are produced and targeted to lysosomes for mitochondrial antigen processing and T cell activation [62]. Other PD-associated genes have also been linked to PINK1/Parkin mitophagy. In particular, mutations in LRRK2 (G2019S and R1441C), which constitute the most common monogenic cause of PD, lead to impaired accumulation of its substrate Rab10 on depolarised mitochondria and diminish PINK1/Parkin dependent mitophagy [17], highlighting a potential general role for mitophagy in pathobiology of PD.

In addition to Parkin's indirect influence on neuronal vulnerability, Parkin also exerts a more direct influence on cell survival through its engagement with the apoptosis machinery. Parkin has been shown to promote cell survival by directly inhibiting the pro-apoptotic activity of the BCL-2 proteins BAX and BAK through degradative and non-degradative mechanisms, whilst converserly promoting cell death by degrading the pro-survival protein MCL-1 [63–65]. The multifactorial influence of PINK1/Parkin in promoting neuronal survival, the prevalence of *PINK1* and *PRKN* mutations in PD, and the well-resolved molecular details of the PINK1/Parkin mitophagy pathway, have prompted a focus on targeting this pathway to treat PD.

## Therapeutic targeting of PINK1/Parkin mitophagy in PD

Pathogenic mutations occur throughout *PRKN* and *PINK1* affecting all domains of the Parkin and PINK1 proteins, respectively [66]. Failure of PINK1/Parkin-mediated mitophagy due to these hypomorphic mutations is linked to the selective death of DA neurons [67]. Furthermore, polymorphisms in *PRKN* and *PINK1*, or reduced activity of Parkin or PINK1, is potentially associated with sporadic PD, suggesting a potential role for this pathway in PD pathophysiology [16,19,20,68–70]. Enhancing PINK1/Parkin mitophagy is thus an attractive strategy to mitigate DA neuron loss in PD patients with PINK1/Parkin loss, but also in sporadic PD. An important step for preclinical validation of PINK1/Parkin-mediated mitophagy activators, and for future translational efforts, is the generation of relevant animal models that display loss of DA neurons and consequent motor deficits which are characteristic of PD. Initial studies on *PINK1* and *parkin* knockout mice identified no significant neurodegeneration or motor deficit [71–73]. However, studies have found that *parkin* knockout mice that were aged for more than 2 years or were progeroid (through accumulation of mitochondrial DNA mutation due to a proofreading defect in DNA polymerase γ) have reported inflammation, neurodegeneration, and motor deficits reminiscent of PD [74,75]. Additionally, *parkin* knockout mice subjected to exhaustive exercise displayed elevated inflammation, although this did not result in neurodegeneration [33]. Remarkably, through triggering an adaptive immune response to cells with damaged mitochondria, intestinal infection of *PINK1* knockout mice with gram negative bacteria was sufficient to provoke striatal DA neurons loss and PD-like motor deficit [61]. These mouse models that phenocopy specific defects in PINK1/Parkin mitophagy, potentially by replicating the genetic defects seen in patients, offer opportunities for a precision medicine approach to treat PD. These proof-of-concept strategies will ultimately drive the development of disease modifying clinical interventions in PD. If successful, this could provide a broader framework to approach the treatment of other neurological conditions.

## Parkin and PINK1 activators

Parkin is a RING-Between-RING E3 ubiquitin ligase that is autoinhibited under basal conditions [76]. The activation of Parkin in response to mitochondrial depolarisation has been resolved in atomic detail over the past decade [41,49,77–81]. Binding of phosphorylated ubiquitin triggers the release of the Parkin N-terminal Ubl domain from the hydrophobic core of the protein. This liberated Ubl can then be phosphorylated by PINK1 and the resulting phospho-Ubl then binds to the Unique Parkin Domain (UPD) of the protein. This then enables the second step of the activation cascade which releases the RING2 domain thereby exposing the catalytic residue, Cys431. With the catalytic cysteine exposed, Parkin is primed to receive a ubiquitin molecule from an E2 ubiquitin-conjugating enzyme, which it can then conjugate to a substrate. Structural resolution of each step of this activation cascade has enabled rational design of molecules that target unique conformations of Parkin to activate the protein in a highly context dependent manner to trigger ubiquitin ligase activity and mitophagy. For example, mutations in Parkin identified in EOPD subjects render the protein inactive due to a blockade in this activation cascade. While mutations that lead to the deletion of exons, or disrupt the zinc-coordinating cysteines may not be reversible, the activity of certain Parkin mutants, such as those in the PINK1 activation domain (e.g. K161N), have been shown to be restored to levels comparable to wild type Parkin though the introduction of compensatory mutations, and so could be rescued by small molecule agonists [78,82,83]. Small molecules that can induce similar conformation changes as these compensatory mutations may be used to restore activity of hypomorphic Parkin in specific EOPD patients. Furthermore, these small molecules provide an attractive alternative to direct activators of the auto-inhibited form of Parkin that may have limited potential and a small therapeutic window [84]. Alternatively, molecules that selectively enhance Parkin activity in the brain may be explored e.g. Rho-associated Protein Kinase 2 (ROCK2) inhibitors [85]. These interventions will provide a rationale for designing drugs that induce mitophagy as a strategy to alleviate PD.

PINK1 levels under basal conditions are restricted through constant protease mediated processing within the mitochondria, followed by proteasomal degradation. Under basal conditions PINK1 is imported into functional mitochondria where its mitochondrial targeting sequence is cleaved (residues 1-34) by the mitochondrial processing peptidase within the mitochondrial matrix. The resultant product is further cleaved at the inner mitochondrial membrane by the mitochondrial proteases PARL to expose PINK1 as an N-end rule substrate and promote its degradation in the cytosol by the proteasome [86,87]. Under oxidative stress (concomitant with mitochondrial damage and loss of mitochondrial membrane potential), PINK1 is stabilised on the OMM at the TOM complex [88,89]. PINK1 expression is also ramped up through transcriptional regulation following the stabilisation of NRF2 (nuclear factor (erythroid-derived 2)-like 2) [90]. Stabilised PINK1 dimerises and transautophosphorylates to activate its ubiquitin kinase activity [47,48]. A particular challenge in the screening of PINK1 activators using phenotypic compound screens in cells is that efforts may identify compounds that function through depolarising mitochondria or an induction of oxidative stress. The recent elucidation of the stepwise mechanism of PINK1 activation can now facilitate the design of targeted screens that focus on identifying agents that modulate specific activation steps in the PINK1 activation cascade, e.g. the autophosphorylation of PINK1. The recent development of artificial intelligence in protein structure prediction will also be a powerful tool to further aid these efforts [48,91,92].

To date, two major classes of PINK1 activators have been described, KTP (kinetin triphosphate) and its analogues [93,94], and niclosamide and its analogues [95]. KTP is an analogue of ATP (N6 furfuryl ATP) with greater catalytic efficiency that enhances activity of recombinant PINK1 and PINK1-mediate mitophagy in cultured cells. It can also rescue defects in mitochondrial morphology and motor deficits in *PINK1*-knockdown flies [93,94,96,97]. KTP was well tolerated in a preclinical rodent model of sporadic PD but did not provide benefit in these models [73]. While this study included *PINK1* knockout rats, the lack of neuronal degeneration or a PD phenotype in these rats precluded an assessment of neuroprotection. Thus, further studies will be required to ascertain the benefit of KTP (and analogues) in PD models that display aberrant PINK1/Parkin mitophagy [73].

Niclosamide, an oral antihelminthic drug, induce reversible mitochondrial depolarisation to enhance PINK1 stabilisation and activity in cultured neurons [95,98]. Gemcitabine, a chemotherapeutic against cancer, has been recently described to potentiate mitophagy through a PINK1-dependent mechanism but independent of Parkin activity [99]. This activity was found to be instead dependent on MUL1, another E3 ubiquitin ligase [99]. While this offers an opportunity for drug repurposing for the treatment of PD, these are yet to be tested in animal models.

More recently described PINK1/Parkin mitophagy activators, identified through high-throughput screening, computational simulation-based screens and other efforts provide attractive avenues for the development of PINK1/Parkin based PD therapeutics [96,97,100] (Table 1). Two related compounds T0466 and T0467, identified through cell based high-throughput screens stimulate mitophagy by inducing PINK1-dependent Parkin translocation to mitochondria [96]. Another small molecule, BC1464, facilitates enhanced PINK1 activity by stabilising full length PINK1 via disrupting its interaction with F-box only protein 7 (FBXO7) [100]. FBXO7 has been implicated in the ubiquitination and degradation of PINK1 and thus targeting this protein–protein interaction may be an additional avenue to enhance PINK1/Parkin mitophagy [100]. Another avenue to boost PINK1/Parkin mitophagy may be via inhibiting kinase activity of LRRK2 using CNS penetrant agents such as GSK3357679A which can correct altered brain mitophagy in a LRRK2 G2019S mouse model [18]. While this benefit is reportedly via enhanced basal mitophagy, there is evidence to suggest this strategy may additionally potentiate PINK1/Parkin mitophagy [17].

**Table 1:** PINK1/Parkin mitophagy activators and their suggested mechanism of action

Compound	Reported mode of action	Model system	Reference
Rho-associated Protein Kinase 2 (ROCK2) inhibitors	Elevated recruitment of hexokinase 2, a positive regulator for Parkin recruitment in depolarised mitochondria	Dopaminergic SH-SY5Y neuroblastoma cells, *Drosophila* model	[85]
GSK3357679A	LRRK2 inhibition	G2019S mouse model	[18]
T0466 T0467	PINK1/Parkin activity dependent, induces Parkin translocation to mitochondria	Dopaminergic neurons, muscle specific PINK1 KO in *Drosophila*	[96]
KTP (kinetin triphosphate) and analogues	ATP analogue with greater kinetic efficiency, enhances PINK1 activity, improves mitochondrial morphology	PINK1 knockdown *Drosophila*, sporadic PD rodent model	[73,93,94,96,97]
Niclosamide and analogues	reversible mitochondrial depolarisation to enhance PINK1 stabilisation and activity	Primary cortical neurons, motor neurons, HeLa cell line	[95,98]
BC1464	Disrupts FBXO7-PINK1 interaction, stabilises full length PINK1, enhances PINK1 activity	MPP^+^ toxicity in SH-SY5Y neuroblastoma cells, mouse primary cortical neurons, PD patient-derived fibroblasts	[100]
Gemcitabine	PINK1/MUL1 dependent mitophagy, Parkin independent	HeLa cell line	[99]
FT3967385	Selective covalent inhibition of USP30	SH-SY5Y neuroblastoma cells	[101]
MF-094	Selective inhibition of USP30 (potentially non-covalent)	isolated mitochondria from C2C12 mouse myoblasts	[102]
15-oxospiramilactone	Inhibition of USP30 activity through interaction with catalytic cystein in the active site	HeLa cell line and mouse embryonic fibroblast cells	[103]
USP30Inh-1, -2 and -3	Inactivation of USP30 via covalent linkage with catalytic cystein within the active site	SH-SY5Y neuroblastoma cells, iPSC-derived dopaminergic neurons and astrocytes, EOPD patient fibroblasts	[104]
Q14 peptide	Allosteric autoinhibition of USP30; interaction with LC3 through its LC-interacting region (LIR) to enhance autophagosome formation	A172 human glioblastoma cell line	[105]

## USP30 inhibitors

USP30 is a de-ubiquitinating enzyme localised on the OMM required for the quality control of proteins being imported into the mitochondria [106,107]. During mitophagy, USP30 opposes the function of Parkin through the removal of ubiquitin from Parkin substrates [55,56,101,104,108,109]. The promise of a treatment avenue from USP30 inhibition stems from preclinical observations that its inhibition can enhance mitophagy in the central nervous system. Knockdown of USP30 in DA neurons of flies rescued the mitophagy deficit and aberrant mitochondrial morphology in flies harbouring pathogenic loss of Parkin or PINK1 and protected against paraquat-induced PD phenotype [56]. In cultured rat and human neurons, pharmacological or genetic inhibition of USP30 enhanced mitophagy [56,104]. Furthermore, pharmacological inhibition of USP30 enhanced phosphorylated (Ser65) ubiquitin in human DA neurons and astrocytes derived from induced pluripotent stem cells, as well as in fibroblasts harbouring Parkin mutations [104]. Various inhibitors of USP30 have been described, e.g. MF-094, 15-oxospiramilactone, FT3967385 [101–103] (Table 1). Some, e.g. FT3967385, have been confirmed to be highly selective for USP30 and are being explored in drug development pipelines for PD and other indications [110].

USP30 has been recently shown to fulfil a fundamental role in regulating protein import at the mitochondrial TOM complex [104]. Whilst this may raise concerns of potential on-target toxicity of pharmacological USP30 inhibitors, mice lacking USP30 are viable with no overt developmental defects [107], suggesting USP30 inhibition could be tolerated. Another potential concern is the off-target effects of current USP30 inhibitors on other proteins, particularly other de-ubiquitinating enzymes [101,107]. Recently a novel peptide (Q14) derived from the transmembrane domain of USP30 has been described that potentiates mitophagy through allosteric autoinhibition of USP30 and though additional interactions with LC3 to stimulate autophagosome formation [105]. So, whilst evaluating USP30 inhibitors for their chronic effects in preclinical animal models is necessary, USP30 inhibition remains an additional strategy to stimulate mitophagy.

## Therapeutic targeting of alternative mitophagy pathways

### BNIP3L/Nix pathway

**Figure 2.**
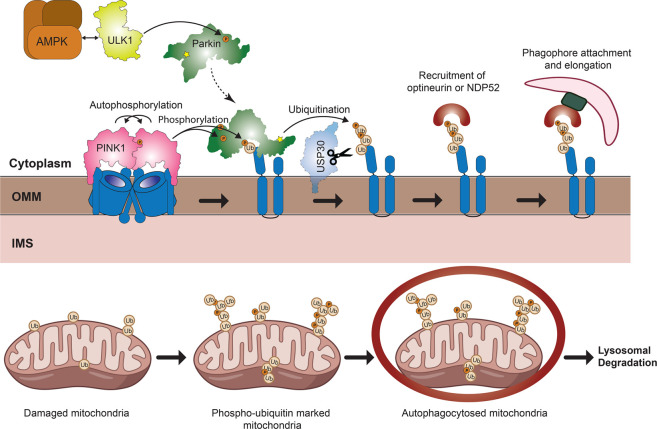
Therapeutic strategies to induce mitophagy. Mitochondrial damage leads to loss of ATP generation and elevation in oxidative stress via generation of reactive oxygen species (ROS) and inflammatory signalling via the release of mitochondrial DNA (mtDNA) as a Danger-Associated Molecular Pattern. This can be mitigated through clearance and recycling of these damaged mitochondria through mitophagy. PINK1/Parkin-dependent mitophagy is the most well studied pathway and inducers of PINK1/Parkin, or inhibitors of USP30, may promoted this process. Alternative mitophagy pathways exist (e.g. BNIP3L/Nix, FUNDC1, iron-chelation) that may function to remove damaged mitochondria including under conditions where PINK1 or Parkin are compromised. Modulating mitophagy thorough one or more of these pathways is emerging as an potential therapeutic strategy in Parkinson's disease. Novel strategies to bypass these mitophagy pathways entirely are now being explored. AUTACs (Autophagy-targeting chimera) are heterobifunctional compounds consisting of a targeting ligand (that binds to a protein or organelle that is to be degraded) and a degradation tag [guanine derivative] that recruits the autophagy machinery. These bifunctional moieties are connected to each other through a flexible ‘linker’, and have been employed in experimental systems to trigger Parkin-independent mitophagy.

While PINK1/Parkin-mediated mitophagy is the most studied mitophagy pathway, especially in the context of PD, pathways that compensate for this process exist and potentially mask the failure of this pathway in certain settings [97] (Figure 2). While subjects carrying homozygous recessive mutations in PINK1 or Parkin develop EOPD, it is important to note that many individuals are asymptomatic until the fourth and fifth decade. In unique circumstances that highlight compensatory mitophagy mechanisms, a subject with homozygous Parkin mutation, and no detectable protein or Parkin activity, exhibited no motor deficit till the seventh decade of life [111,112]. This individual harboured elevated levels of Nix, a component of the BNIP3L/Nix mitophagy pathway. This pathway is commonly induced in response to hypoxia and programmed mitochondrial clearance during erythrocyte development yet provides an alternative target to enhance mitophagy in the context of PD [113,114]. Elevation of Nix genetically or pharmacologically compensates for PINK1 or Parkin loss, restoring mitophagy in cultured fibroblasts [112]. As Nix is also reported to be a potential substrate of Parkin [115], enhanced Nix may promote Parkin-dependent mitophagy when this pathway remains intact, such as in cases of sporadic disease or when Parkin mutations are hypomorphic and do not completely ablate Parkin activity.

### FUN14 domain-containing protein 1 (FUNDC1) pathway

FUNDC1 is a OMM protein that can recruit microtubule-associated protein light chain 3 (LC3) independent of Parkin-mediated ubiquitination to instigate mitophagy. FUNDC1 possesses an LC3 Interacting region (LIR), which, upon hypoxic stress, associates with LC3 following dephosphorylation of Ser13 and Tyr18 by the phosphatase PGAM5 and an unknown phosphatase, respectively [116,117]. The simultaneous phosphorylation of Ser17 by ULK1, further enhances the interaction of FUNDC1 with LC3 [116]. This process is fine-tuned by the mitochondrial E3 ubiquitin ligase MARCHF5. MARCHF5 directly interacts with FUNDC1 resulting in its degradation and a slowing of the mitophagy process. Knockdown of MARCHF5 blocks the degradation of FUNDC1 and sensitises mitochondria to mitophagic stressors [118]. Hence, the modulation of MARCHF5/FUNDC1 axis may provide additional ways to enhance mitophagy to remedy PD. MARCHF5 has also been reported to potentiate Parkin activity by providing the necessary ubiquitin landscape for Parkin recruitment [119], hence MARCHF5 may also be a target to promote Parkin-dependent and -independent mitophagy.

### Iron chelation-induced mitophagy

How iron deprivation induces mitophagy is yet to be clearly defined, but it is associated with stabilisation of the hypoxia responsive transcription factor HIF1α [120,121] and is independent of Parkin [120,121]. This mode of mitophagy is coupled with elevated mitochondrial ferritin and its interaction with Nuclear Receptor Coactivator 4 (NCOA4). NCOA4 is the autophagic cargo receptor that is typically enriched in autophagosomes and is responsible for turnover of the cytosolic iron sequestration protein, ferritin [120–122]. Interestingly, mitochondrial ferritin is expressed highly in the brain, is inducible by HIF1α, and prevents mitochondrial damage and neuronal apoptosis in the 6-OHDA neurotoxin model of PD [123–125]. As iron chelators are already in clinical use for other indications and may facilitate neuroprotection though additional mechanisms, such as mitigating oxidative stress, extinguishing oxidative cell death pathways, and mitigating age-associated chronic sterile inflammation associated with neurodegeneration [30,123–127], iron deprivation induced mitophagy is an attractive avenue for PD therapeutics. Deferiprone, an orally bioavailable iron chelator approved for thalassemia that can cross the blood-brain-barrier, induces Parkin-independent mitophagy [120,121]. Deferiprone induced mitophagy was recently described to be dependent on the mitochondrial fission 1 (Fis1) protein [128]. Deferiprone treatment led to the stabilisation of SENP3 a protease that cleaves the post-translational SUMOylation (at Lys149) of Fis1 facilitating its mitochondrial localisation and stress-induced mitophagy [128]. Furthermore, Deferiprone displayed a good safety profile and an indication of slower disease progression in two published human clinical trials in PD (NCT00943748, NCT01539837) [129,130]. Based on these positive trial outcomes, deferiprone has progressed to two large phase II clinical trials to evaluate the effects of iron chelation on motor and non-motor symptoms in PD (NCT02655315, active; NCT02728843, concluded). The results of these trials are awaited but understanding the contribution of mitophagy induction for any observed clinical benefit will support the development of other mitophagy-inducing drug candidates.

## AUTACs: bypassing mitophagy machinery with small molecules

AUTACs (Autophagy-targeting chimeras) are small molecule degraders that high-jack the autophagy machinery to degrade proteins and even whole organelles [131–133]. The first class of these degraders are based on the ubiquitin-dependent autophagic clearance of group A *Streptococcus* (GAS). While the E3 ubiquitin ligase involved is yet to be identified, S-guanylation of the bacterial surface by the endogenous nucleotide (8-nitro-cGMP) promotes GAS ubiquitination via Lys63 linkage to promote xenophagy [132,133]. Based on this observation, membrane-permeable AUTACs were developed that comprised a degradation tag (guanine derivative) connected to a substrate targeting ligand by a flexible small linker (Figure 2). Whilst AUTAC-mediated autophagy is ubiquitin-dependent, it does not require Parkin or PINK1, allowing the autophagic machinery to bypass potential dysfunction in the PINK1/Parkin mitophagy pathway. In a proof-of-concept study, the mitochondria-targeted AUTAC4 promoted the degradation of fragmented mitochondria and restored mitochondrial membrane potential and ATP generation in fibroblasts established from a Down syndrome patient [133]. Although the utility of these compounds has yet to be tested in pre-clinical models of PD, AUTACs represent a potentially powerful new class of compounds to therapeutically stimulate mitophagy.

## Conclusion

Dysfunctional mitochondria and impaired mitophagy are emerging as potentially cardinal features of PD. Detailed understanding of the molecular events that regulate mitophagy is thus critical for the development of therapies to repair or restore this process when it goes awry. The molecular control of PINK1/Parkin mitophagy has been resolved in great structural detail, potentially paving the way for targeted disease-modifying therapies to treat EOPD patients that harbour genetic defects in *PINK1/PRKN*. Alternative mitophagy pathways could also offer therapeutic avenues to a wider cohort of patients once proof-of-concept is established. To aid these precision medicine approaches, mouse models carrying pathogenic genetic defects that cause EOPD and phenocopy PD symptoms are essential for preclinical screening of mitophagy modulators and for understanding the contribution of mitophagy in the pathophysiology of PD.

Recent technological breakthroughs such as machine learning approaches which allow for structural resolution of the entire human proteome are emerging together with new strategies such as AUTACs, that can be designed to degrade specific biological targets. These will undoubtedly benefit the search for disease-modifying therapies in neurodegenerative conditions like PD that are still largely considered proteinopathies. The fundamental question around the utility of modulating mitophagy in PD will be ultimately resolved through trialling these preclinical strategies in patients that are stratified based on their genetics and/or biology to be the most likely to benefit.

## Perspectives

- Impaired mitophagy is a feature of familial (genetic) and sporadic Parkinson's disease (PD) and enhancing mitophagy may offer disease-modifying potential.
- The resolution of mitophagy processes in molecular detail will enable precision medicine approaches to treat PD and potentially other complex neurodegenerative conditions.
- Innovative technologies such as AUTACs provide novel strategies for the development of mitophagy-targeted therapeutics.

## Open Access

Open access for this article was enabled by the participation of University of Melbourne in an all-inclusive *Read & Publish* agreement with Portland Press and the Biochemical Society under a transformative agreement with CAUL.

## Abbreviations

6-OHDA: 6-hydroxydopamine
ATG8: Autophagy-related protein 8
AUTAC: Autophagy-Targeting Chimera
BNIP3L: BCL2/Adenovirus E1B 19 KDa Protein-Interacting Protein 3-Like
Cys: Cysteine
DA: Dopaminergic
DFCP1: Double FYVE-containing protein 1
EOPD: early-onset PD
FUNDC1: FUN14 domain-containing protein 1
GAS: group A *Streptococcus*
HIF1α: Hypoxia-inducible factor 1-alpha
KTP: kinetin triphosphate
LC3: microtubule-associated protein light chain 3
LIR: LC3 Interacting region
Lys: Lysine
MARCHF5: Membrane Associated Ring-CH-Type Finger 5
MPTP: 1-methyl-4-phenyl-1, 2, 3, 6-tetrahydrodropyridine
NCOA4: Nuclear Receptor Coactivator 4
NDP52: Nuclear dot protein 52 kDa
NRF2: nuclear factor (erythroid-derived 2)-like 2
OMM: Outer Mitochondrial Membrane
PARL: Presenilins-associated rhomboid-like protein
PD: Parkinson's disease
PGAM5: Phosphoglycerate mutase 5
PINK1: PTEN-induced putative kinase 1
PRKN: Parkin
ROS: Reactive oxygen species
Ser: Serine
SN: *Substantia nigra*
SNpc: *SN pars compacta*
TOM: Translocase of the Outer mitochondrial Membrane
Tyr: Tyrosine
Ubl: ubiquitin-like domain
ULK1: Unc-51 Like Autophagy Activating Kinase 1
UPD: Unique Parkin Domain
USP30: Ubiquitin Specific Peptidase 30
WIPI1: WD Repeat Domain Phosphoinositide Interacting 1
